# Contralateral recurrence of fallopian tube torsion: A case report

**DOI:** 10.1016/j.crwh.2021.e00307

**Published:** 2021-03-16

**Authors:** Daniil Khaitov, Nagaraj Gabbur

**Affiliations:** a Resident in Obstetrics and Gynecology, Northwell Health, Department of Obstetrics and Gynecology, Manhasset, NY, USA; b Residency Program Director of Obstetrics and Gynecology, Zucker School of Medicine at NorthShore, Long Island Jewish Hospital, USA

**Keywords:** Fallopian tube torsion, Paraovarian cyst

## Abstract

Unilateral lower quadrant pain is a common presenting complaint in the emergency room with a wide differential. It is important to consider fallopian tube torsion in the differential, especially in premenopausal women, as fertility-sparing detorsion, especially in a woman with a previous salpingectomy or other fertility-affecting surgery, is essential. This case report is of a 25-year-old woman with worsening left lower quadrant abdominal pain over 24 h found to have an extraovarian cystic mass. When taking into consideration the patient with a history of contralateral fallopian tube torsion secondary to a paraovarian cyst, now presenting with left lower quadrant abdominal pain and a cystic extraovarian mass, immediate laparoscopic evaluation was warranted. Immediate intervention revealed an isolated fallopian tube torsion and resulted in surgical preservation of fertility.

## Highlights

• Recurrent paratubal cyst resulting in isolated fallopian tube torsion
• Immediate fallopian tube detorsion as fertility sparing procedure
• Gross necrotic appearance of adnexa does not require salpingectomy/oopherectomy.

## 1 Introduction

Isolated fallopian tube torsion is reported to be as rare as 1 in 1.5 million [1]. It may also be difficult to diagnose on imaging as there are no pathognomonic findings consistent with this diagnosis. This case describes the signs and symptoms, and how the importance of having a history of a fallopian tube torsion should increase the clinician's suspicion for recurrence, even though rare.

## 2 Case Presentation

A 25-year-old nulliparous woman presented with 24 h of increasing left lower quadrant pain, worsened with movement and temporarily relieved with narcotic medications given by the emergency room provider. She had a past medical history of PCOS and a surgical history of a right salpingectomy secondary to a right paraovarian cyst resulting in an isolated necrotic torsed fallopian tube at 13 years of age.

Physical exam revealed an obese female with normal vital signs. Abdominal exam was significant for left lower quadrant pain on deep palpation, with no rebound or guarding present. Bimanual examination revealed similar pain at left adnexa but otherwise benign with no vaginal bleeding, cervical motion tenderness or otherwise palpable masses. Of note, the physical examination was performed after patient had already received 975 mg of acetaminophen, 600 mg of ibuprofen and three doses of 2 mg morphine by emergency room providers.

Laboratory evaluation was significant only for a white blood cell (WBC) count of 12.85 × 10^9^/l with an otherwise normal complete blood count, comprehensive metabolic profile and urinalysis. Transvaginal and transabdominal ultrasound revealed a simple left adnexal cyst 1.5 × 1.2 × 1.4 cm and a 4 × 3.8 × 4.1 cm mildly complex cyst, both of which appeared to be extraovarian. Arterial and venous waveforms were documented for both ovaries. Fluid in the endocervical canal was also noted.

**Fig. 1:**
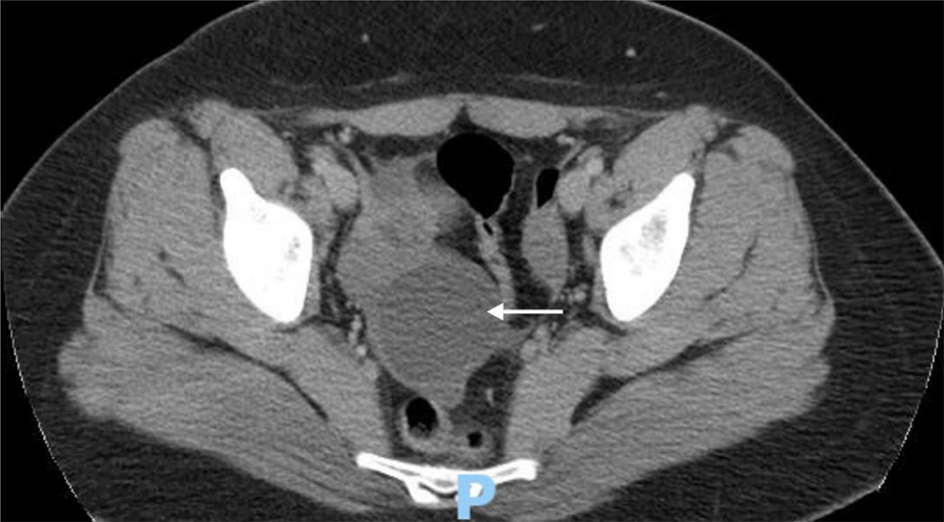
CT abdomen/pelvis with IV contrast demonstrates right adnexal cyst. Ovary in this image unable to be visualized.

The patient's surgical history was significant for a similar presentation to the emergency department at 13 years of age. Upon review of the medical record that was available, a nurse at triage noted the patient was complaining of right lower quadrant pain that worsened over the day and stated that the patient was “doubled over in triage”. Unfortunately, no provider note could be found regarding physical examination and history of presenting illness. A CT examination of the abdomen and pelvis had revealed a 5.1 × 4.4 cm right ovarian cyst with trace amount of surrounding fluid (Fig. 1). Otherwise, the left adnexa and uterus were normal. Review of the operative note for the procedure in 2008 revealed a 6x6cm right adnexal structure that appeared to be torsed, blue and hemorrhagic and included a dilated right tube. A salpingectomy was then performed without complication. The right ovary, left ovary and tube at that time were noted to be grossly normal.

**Fig. 2:**
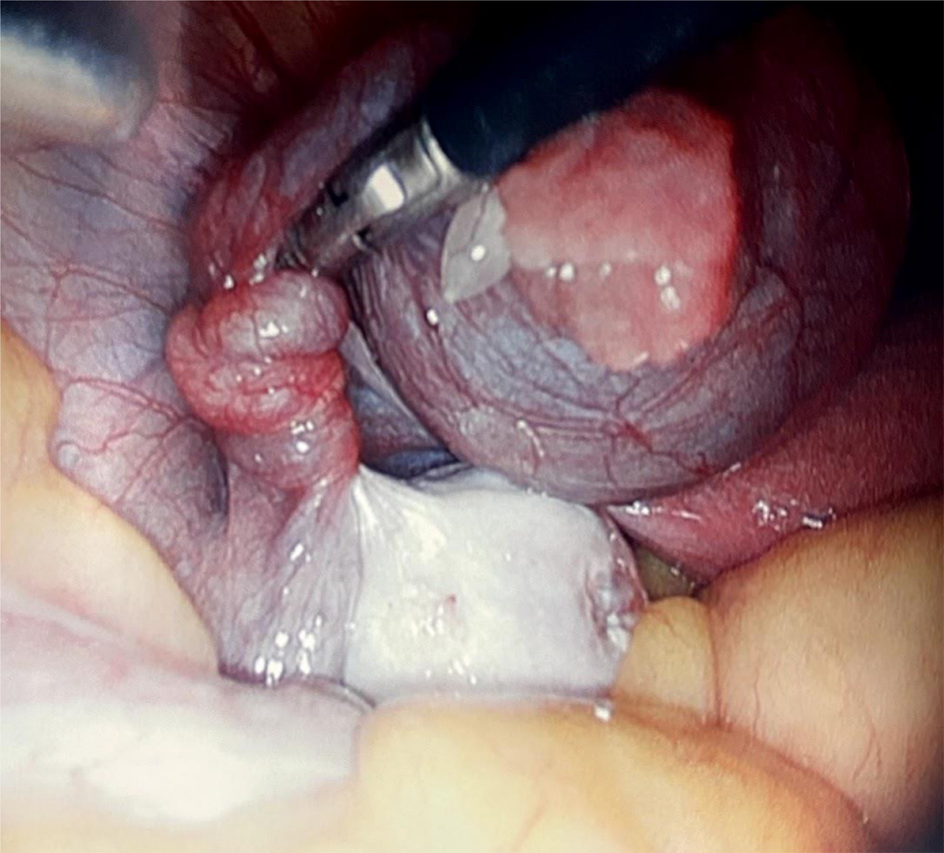
Laparoscopic view of the left fallopian tube torsed over four times with no signs of necrosis, a paratubal cyst and a grossly normal left ovary.

The decision was made to take the patient to the operating room for a diagnostic laparoscopy as there was enough clinical suspicion for either an ovarian, adnexal or tubal torsion based on the physical exam, ultrasound finding of extraovarian cysts and the patient's significant surgical history. Laparoscopically, a left-sided 4 cm paratubal cyst was removed and a left fallopian tube without signs of necrosis was de-torsed four times in a clockwise fashion (Fig. 2). The ovaries bilaterally were grossly normal. Surgical pathology two weeks later revealed a simple paratubal cyst. No frozen specimen was sent intraoperatively as malignancy was of low concern.

## 3 Discussion

This case shows the importance of having a low index of suspicion as well as an emergent plan to action, especially with regard to preservation of fertility for a suspected tubal torsion. Even though the incidence of an isolated tubal torsion is 1 in 1.5 million [2,3], to have a repeat occurrence on the contralateral side, likely unrelated to the previous torsion as diseased pathology was removed in full, is even more rare.

This patient, as in other case reports, presented symptoms that were vague and nonspecific. Pelvic pain and nausea were the main symptoms and were also the most common symptoms in other cases of isolated tubal torsion [4].Ultrasound findings showed an extra-ovarian cyst that was later found to be paratubal on laparoscopy. It has been well reported that a paraovarian cyst is an extrinsic factor associated with tubal torsion [2]. In this case, the presenting symptoms with an adnexal cyst at the very least prompted a laparoscopic evaluation, which is considered the best diagnostic and therapeutic approach [3]. More important, emergent evaluation was vital in preserving the patient's only remaining fallopian tube. Due to nonspecific presenting symptoms and delay to surgical intervention, case reports have shown the inability to preserve fertility by requiring salpingectomy versus detorsion [4,5].

This patient experienced both outcomes regarding fertility-sparing surgery. At 13 years old, based on the operative report, laparoscopic gross evaluation of the right fallopian tube deemed it to be unsalvageable. Factors that have been found to be correlated to adnexal necrosis were onset of symptoms over 10 h prior to surgery and age > 40 [5]. Fortunately for her second time, even though the interval between onset of symptoms and surgery was approximately 24 h, she able to have her fallopian tube remain in situ as no signs of necrosis were present. Moreover, new data on ovarian and adnexal torsion show that even if an ovary appears necrotic, detorsion may still provide reperfusion and prevent the need for oophorectomy [3]. Therefore, the decision for salpingectomy versus detorsion is multi-factorial but, with extrapolation from recent adnexal torsion studies, detorsion should be attempted to preserve fertility. Due to the rarity of this condition, post-operative follow-up with repeat imaging after detorsion has not been recorded.

To conclude, isolated tubal torsion is a rare phenomenon but can have significant consequences for a woman's future fertility. Awareness of this condition is important and prompt laparoscopic intervention can help preserve fertility. Attempt at detorsion even if the fallopian tube appears necrotic should be performed in select cases.
